# High-Density Capacitive Energy Storage in Low-Dielectric-Constant Polymer PMMA/2D Mica Nanofillers Heterostructure Composite

**DOI:** 10.3390/molecules29194671

**Published:** 2024-10-01

**Authors:** Sumit Bera, Rukshan Thantirige, Sujit A. Kadam, Anirudha V. Sumant, Nihar R. Pradhan

**Affiliations:** 1 Department of Chemistry, Physics and Atmospheric Science, Jackson State University, 1400 John R. Lynch Street, Jackson, MS 39217, USA; 2 Center for Nanoscale Materials, Argonne National Laboratory, 9700 South Cass Avenue, Lemont, IL 60439, USA

**Keywords:** PMMA, 2D materials, mica, polymer composite, energy density, polarization

## Abstract

The ubiquitous, rising demand for energy storage devices with ultra-high storage capacity and efficiency has drawn tremendous research interest in developing energy storage devices. Dielectric polymers are one of the most suitable materials used to fabricate electrostatic capacitive energy storage devices with thin-film geometry with high power density. In this work, we studied the dielectric properties, electric polarization, and energy density of PMMA/2D Mica nanocomposite capacitors where stratified 2D nanofillers are interfaced between the multiple layers of PMMA thin films using two heterostructure designs of the capacitors, PMMA/2D Mica/PMMA (PMP) and PMMA/2D Mica/PMMA/2D Mica/PMMA (PMPMP). The incorporation of a 2D Mica nanofiller in the low-dielectric-constant PMMA leads to an enhancement in the dielectric constant, with ∆ε ~ 15% and 53% for PMP and PMPMP heterostructures at room temperature. Additionally, a significant improvement in discharged energy density was measured for the PMPMP capacitor (U_d_ ~ 38 J/cm^3^ at 825 MV/m) compared to the pristine PMMA (U_d_ ~ 9.5 J/cm^3^ at 522 MV/m) and PMP capacitors (U_d_ ~ 19 J/cm^3^ at 740 MV/m). This excellent capacitive and energy storage performance of the PMMA/2D Mica heterostructure nanocomposite may inform the fabrication of thin-film, high-density energy storage capacitor devices for potential applications in various platforms.

## 1. Introduction

The increasing demand for energy storage devices with ultra-high capacity and efficiency has sparked significant research interest in energy storage materials such as lithium-ion batteries, sodium-ion batteries, and dielectric capacitors [1,2,3,4,5]. Dielectric capacitors present compelling advantages over lithium-ion and sodium-ion batteries, including rapid charging, an extended lifespan, and enhanced efficiency, thereby attracting significant attention in electrostatic energy storage research [6]. Polymer dielectrics are the most favored material for the fabrication of electrostatic capacitors due to their efficient energy storage, high dielectric strength, compactness, thermal stability, and cost effectiveness. However, their low energy density, efficiency, and operating voltage limit the application of polymer materials in practical devices [7,8,9]. The incorporation of various types of 2D filler materials within polymer matrices has been a conventional method for overcoming the low discharged energy density (U_d_) and breakdown strength (E_BD_) of the polymer matrices [9].

Several dielectric polymers, such as polyvinylidene fluoride (PVDF), PVDF-TrFE-CFE, poly(methyl methacrylate) (PMMA), and polyimides (PIs), are commonly used in the fabrication of nanocomposite matrices using various nanofillers, such as hexagonal boron nitride (h-BN), barium titanate nanosheet, Mica, titanium dioxide (TiO_2_), aluminum oxide (Al_2_O_3_), molybdenum disulfide (MoS_2_), graphene oxide (GO), reduced graphene oxide (r-GO), MXene, etc. [9,10,11,12,13,14,15,16,17,18,19]. Discharged energy density (U_d_), efficiency (η), and breakdown strength (E_BD_) are the three critical parameters that determine the figure of merit of a capacitor device. These parameters are intrinsically linked with the dielectric polarization at the interface of the polymer and nanofiller entities and how the interface properties change as a function of electric field, temperature, and filler content and configuration. Thus, having a deeper understanding is essential to exploring these composite materials for their potential applications.

The discharged energy density of a dielectric material can be obtained using the area under the electric displacement vs. electric field loop (D-E loop) during discharging. For a linear dielectric material, the maximum discharged energy density of a capacitor $U_{d\_max}$ = $\frac{1}{2}\varepsilon_{r}\varepsilon_{0}{E_{BD}}^{2}$, where $\varepsilon_{r}$ is the dielectric constant of the material or polymer composites and E_BD_ is the maximum electric field that the capacitor can sustain before the leakage current increases. Thus, $\varepsilon_{r}$ and E_BD_ are the two essential parameters in the optimization of U_d_. However, simultaneously enhancing both U_d_ and E_BD_ is still a challenging task, as E_BD_ degrades with the increasing behavior of $\varepsilon_{r}$ [20]. A concurrent improvement in dielectric properties along with ultra-high energy density was observed with the incorporation of Mica in a high-dielectric-constant PVDF polymer [10]. Thin-layer Mica has a high band gap [3–4 eV] and, along with its two-dimensional structure, offers a significant surface area for interaction with the polymer matrix, resulting in improved mechanical strength, thermal stability, and electrical insulation properties in nanocomposites [10]. Furthermore, the fabrication of multilayer polymer nanocomposites originates interfacial polarization, which contributes significantly to an increase in the value of U_d_ and E_BD_ [21,22,23,24]. This work encompasses both approaches: integrating 2D Mica into the PMMA polymer and fabricating a Mica–PMMA multilayer nanocomposite device using a layer-by-layer method where 2D fillers are aligned in a stratified geometry within the thin film of polymer layers. A significant enhancement in the dielectric constant of up to 55% was observed in the PMPMP devices. Moreover, an enhancement of U_d_ ~ 37.5 J/cm^3^ at 825 MV/m with higher η ~ 93% and E_BD_ ~ 865 MV/m was estimated in a multilayered stack of polymer and a 2D filler device.

## 2. Results and Discussion

**Figure 1:**
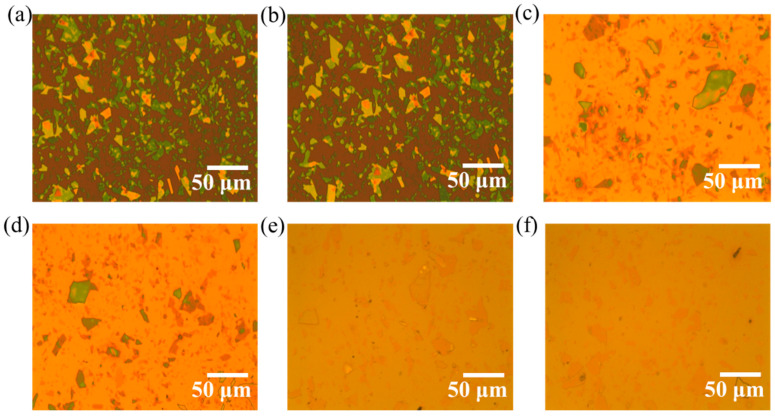
Optical images. (**a**,**b**) Distribution of 2D Mica sheets exfoliated onto Si/SiO_2_, (**c**,**d**) PMMA spin coated on the Mica-exfoliated substrates, (**e**,**f**) PMP heterostructure after annealing at 120 °C for 3 h in vacuum.

**Figure 2:**
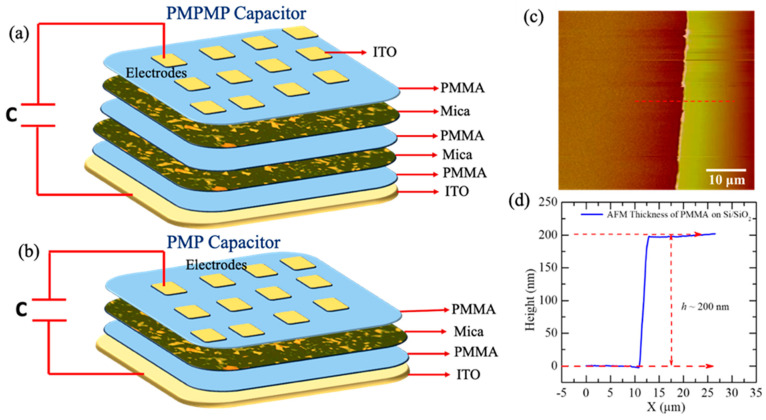
(**a**,**b**) Schematic of PMP and PMPMP heterostructure capacitors. PMP capacitor contained one layer of exfoliated Mica fillers and PMPMP capacitor contained two layers of Mica fillers. (**c**) AFM topography images of PMMA film showing cross-sectional mark. (**d**) Height variation in corresponding PMMA film perpendicular to cross section.

The optical images after exfoliation onto Si/SiO_2_ display 2D Mica flakes of different thicknesses, sizes, and orientations in the 2D plane of the substrate are shown in Figure 1a,b. These flakes were mechanically exfoliated using a scotch-tape method and transferred onto the clean SiO_2_ substrate. The distribution of the flakes is non-uniform across the substrate, which is generally observed in the mechanically exfoliated technique. Figure 1c,d depict the optical images of PMMA-coated Mica flakes on Si/SiO_2_, confirming that the 2D flakes remain intact on the substrate after spin coating. In Figure 1e,f, the surface of the PMP capacitor reveals the integration of 2D flakes between two layers of polymer films, highlighting their incorporation. The PMP and PMPMP capacitors are shown schematically in Figure 2a,b respectively. The thickness of a single PMMA layer was measured as 200 nm using the AFM tapping mode, recording the cross section of the polymer after making a scratch on the film, as shown in Figure 2c. Figure 2d depicts the height profile of the film thickness measured across the scratch, showing an average of 200 nm thick. The total thickness of the PMP devices is 400 nm, while the PMPMP devices have a total thickness of 600 nm. We measured the dielectric constant of pure PMMA, PMP, and PMPMP capacitors to understand the role of nanofillers within the polymer matrix.

**Figure 3:**
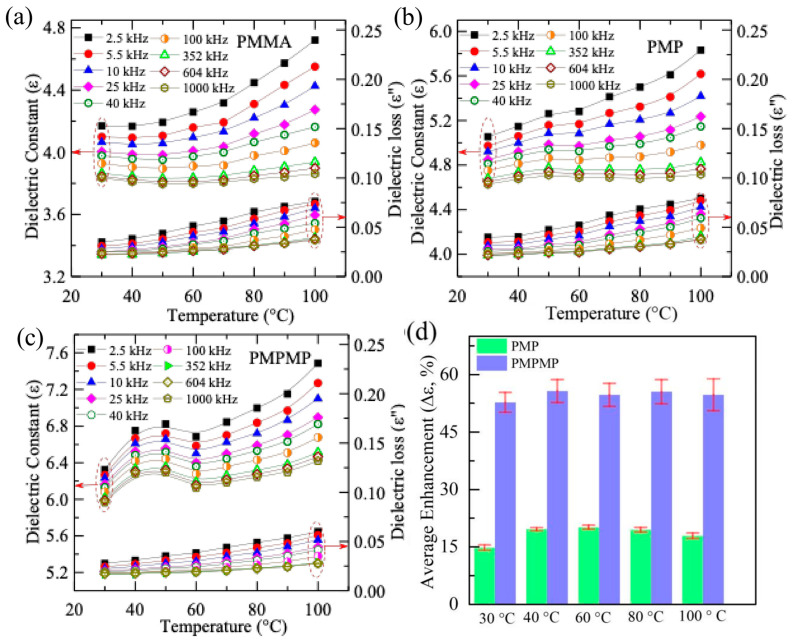
(**a**) Temperature-dependent dielectric constant and dielectric loss of PMMA (**a**), PMP (**b**), and PMPMP (**c**) at different constant frequencies in the range between 2.5 kHz and 1 MHz. (**d**) Histogram showing the enhancement of the dielectric constant (∆ε) of PMP and PMPMP with respect to pure PMMA at a constant frequency of 10 kHz and different temperatures.

Figure 3a–c show the variation in the dielectric constant and loss tangent of the polymer nanocomposites between 30 and 100 °C, while the frequency varies from 100 Hz to 7 MHz. The dielectric constant gradually decreases with increasing frequency throughout the temperature range. As the frequency increases, the dielectric constant of polymer nanocomposites decreases due to the weakening of the polarization components. At higher frequencies, the dipoles have insufficient time to align with the direction of the electric field, resulting in a lower dielectric constant value [25]. The dielectric constant of the pure PMMA polymer varies between 4.5 at room temperature, 30 °C, and 5.1 at 100 °C. The PMP shows an enhancement of 15% (ε ~ 6.1) and 19% (ε ~ 6.5) at the respective temperatures 30 °C and 100 °C, while the PMPMP exhibits more enhancements of 54% (ε ~ 7.1) and 56% (ε ~ 7.5), shown in Figure 3d. The low enhancement of the PMP is probably due to the low loading of nanofillers compared to the PMPMP capacitors, where the two layers of Mica are stacked in heterostructure geometry. This enhancement is also lower than that of the PVDF/2D Mica heterostructure capacitor reported recently [10,26]. The polarization at the interface between PMMA, a low dielectric polymer, and Mica could be lower, which can be one of the interesting topics to explore using theoretical studies.

The enhancement of the dielectric constant in PMP and PMPMP devices can also be attributed to the effect of interfacial polarization occurring at the polymer–polymer interface and the Mica–polymer interface of the multilayer nanocomposite devices [21,22,23,24]. The phenomenon of dielectric constant enhancement can be analyzed using series capacitance models of two dissimilar dielectric materials of different thicknesses and dielectric constants. The dielectric constant (ε) of two different dielectric materials (here, PMMA and Mica) of dielectric constants of $\varepsilon_{1}$,$\;\varepsilon_{2}$ and thicknesses of $d_{1},\;d_{2}\;with$ heterojunction can be obtained by the series capacitor model, $\varepsilon_{=}\;\frac{\varepsilon_{1}\;\varepsilon_{2}(d_{1}+d_{2})}{\left(d_{2}\varepsilon_{1}+d_{1}\varepsilon_{2}\right)}$. The average thickness of the 2D Mica flakes is 20 nm, as reported in our previous work [10]. Taking $\varepsilon_{1}$ ~ 4.5 (dielectric constant of PMMA), $\varepsilon_{2}$ ~ 7 (dielectric constant of Mica), and $d_{1}\;$= 200 nm, $d_{2}\;$= 20 nm, we obtain ε~4.65, which is higher than the PMMA [27]. Hence, the presence of multiple layers of different dielectric materials justifies the enhancement of the overall dielectric constant in the PMPMP bilayer heterostructure.

At room temperature, the dielectric losses of PMMA, PMP, and PMPMP at 1 kHz are 0.035, 0.038, and 0.028, respectively. Hence, the integration of one layer of Mica in the PMP device does not significantly impact the dielectric loss of the polymer nanocomposite, while the addition of a second layer of PMMA–Mica reduces the loss factor in the PMPMP heterostructure, as shown in Figure 3a–c.

In general, there are two types of losses that play a role in a dielectric material: (a) conduction loss, which arises from the collision, trapping, and recombination of free charges in charged capacitors; and (b) polarization loss, which occurs due to the displacement of bound charges during polarization [28]. The presence of an additional interface and insulating Mica layer in the PMPMP device results in a drop in dielectric loss [24]. The presence of multiple interfaces deviates the electric trees along the interface plane, while the insulating Mica weakens the propagation of the electric tree, restricting the motion of the charge carrier [29,30,31,32,33]. The enhanced dielectric constant and reduced loss factor in the PMP and PMPMP devices point toward improved performance.

**Figure 4:**
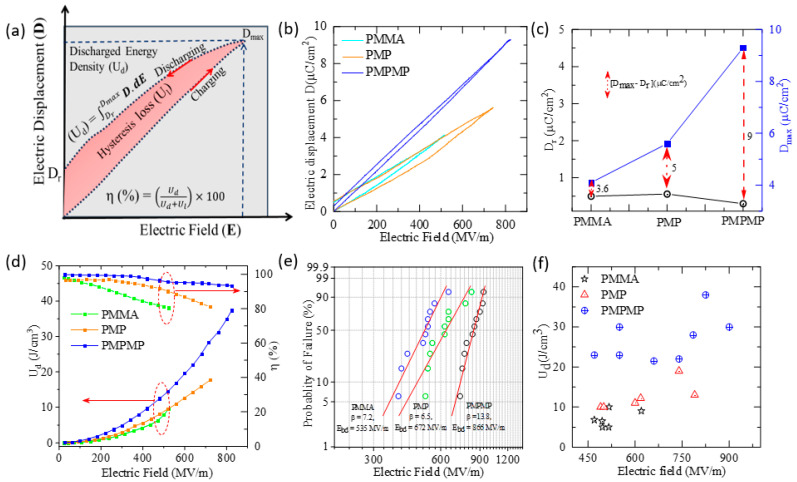
(**a**) Schematic diagram outlining the charging and discharging cycles of a D-E loop, including D_r_, D_max_, and discharged energy density (U_d_), (**b**) field-dependent variation in electric displacement (D) of PMMA, PMP, and PMPMP, (**c**) remnant electric displacement (D_r_) and maximum electric displacement (D_max_) of the polymer and polymer nanocomposites, (**d**) variation in discharged energy density (U_d_) and efficiency (η) with electric field, (**e**) Weibull probability fit showing the calculated breakdown strength (E_BD_) and shape parameter (β), (**f**) maximum discharged energy density (U_dmax_) of different capacitors of the polymer and polymer nanocomposites with electric field.

Energy density is a key factor in energy storage devices [10,26,34]. We measured the polarization vs. applied electric field to elucidate the energy storage property of the capacitors. In contrast, Figure 4a shows the schematic of the typical P-E loop of a ferroelectric materials capacitor with hysteresis loss (indicated by the pink color), discharge, and charge energy densities. One would expect low hysteresis loss in non-ferroelectric polymers compared to the ferroelectric materials. Figure 4b shows the electric field variation in the electric displacement (D) of PMMA, PMP, and PMPMP heterostructure capacitors at room temperature. The variation in D with the electric field shows a trend of higher polarization and high field sustainability in the nanocomposites with the addition of a 2D Mica filler. This enhancement in field sustainability is also a phenomenon observed in 2D material-based gas sensors [35,36], where the incorporation of layered nanomaterials improves both sensitivity and stability. The energy densities in the charging and discharging cycles are referred to as U_e_ and U_d_, which are basically the areas under the charging and discharging cycles in the D-E loop graph, as shown in the schematic in Figure 4a. The discharged energy density can be calculated as U_d_ = $\int_{D_{r}}^{D_{max}}D.dE$, and the area of the D-E loop refers to the hysteresis loss (U_l_). The charged energy density U_e_ = U_d_ + U_l_. The ratio of the energy densities at a particular electric field is referred to as efficiency, η (%) = $\frac{U_{d}}{U_{e}}\times 100.$ In optimizing a D-E loop for ultra-high U_d_ and η, it is necessary to boost the maximum electric displacement (D_max_) and minimize the remnant electric displacement (D_r_) while maintaining a robust electric field sustainability. The PMMA exhibits a low value for both D_r_ and D_max_, and high electric field sustainability varies between 400 and 750 MV/m. The capacitors also show minimum hysteresis loss. These combinations result in a moderate U_d_ of 8 to 15 J/cm^3^, while efficiency remains relatively high up to 80% [33,37,38,39,40]. Hence, when obtaining a higher D_max_ to elevate the U_d_, it is essential to improve the polarization of the polymer. Our capacitor shows the enhanced polarization by introducing the 2D nanofillers between the polymer films. It is observed that the remnant electric displacement (D_r_) of the PMMA and PMP capacitor devices is close to ~ 0.5 µC/cm^2^, while it is reduced to 0.3 µC/cm^2^ for the PMPMP device. The maximum electric displacement (D_max_) increases significantly with the loading of Mica layers between the polymer layers, as shown in Figure 4c. The D_max_−D_r_ increases with the addition of Mica layers from 5 C/cm^2^ in the PMP to 9 C/cm^2^ in the PMPMP. This implies a reducing loss feature from PMP to PMPMP capacitors, corroborating the dielectric loss versus temperature variation observed earlier. Figure 4d shows the dynamics of U_d_ and η of the PMMA, PMP, and PMPMP nanocomposites with the variation in electric field (E). The discharged energy density of pure PMMA was calculated as U_d_ ~ 9.5 J/cm^3^ at 522 MV/m, with η ~ 80.5%. The U_d_ and η at the same electric field for the PMP capacitor are 9.6 J/cm^3^ and 90%, and for the PMPMP they are 14 J/cm^3^ and 95%, respectively. The energy density of pure PMMA is similar to the reported values in the literature [39].The highest energy density and efficiency of the PMP device were calculated as U_d_ ~ 17.7 J/cm^3^ and η ~ 81.2% at 720 MV/m. The addition of a second Mica–PMMA layer to the PMP capacitor enhances the energy density and efficiency to 37.35 J/cm^3^ and 93% at the highest measured electric field of 825 MV/m, as shown in Figure 4d. Polymer-based dielectrics are widely used as insulation materials for high-voltage applications due to their intrinsic high breakdown strength. Beyond that, these materials, with suitable filler combinations, can possess high charge accumulation but exhibit high resistance to charge percolation by providing ordered traps and scattering centers to mobile charges and by increasing path tortuosity for carrier treeing propagation. We measured the breakdown voltages of the capacitors and used a Weibull probability distribution P(E) = $1-{exp}^{{-\left(\frac{E}{E_{BD}}\right)}^{\beta }}$ fitting to obtain the breakdown fields (E_BD_) of these polymer nanocomposite film capacitors, where P is the cumulative probability of failure, E is the electric field at dielectric failure, E_BD_ is the breakdown strength, which is the dielectric strength at 63.2% probability of failure, and β is a shape parameter. The value of β in a Weibull fit indicates the shape or randomness of data points in the distribution. Having a larger β implies a narrow distribution and higher consistency of breakdown strength of the capacitor. A total of 10 data points were collected from various capacitors to fit the Weibull probability distribution and determine the exact breakdown strength. The PMMA shows high breakdown strength compared to other polymer matrices, but the low polarization limits the discharged energy density (U_d_) [16,33,38,41]. Zhang et al. reported a maximum U_d_ ~ 14.6 J/cm^3^ with η ~ 73.47% at E ~ 809 MV/m for the PMMA film [4]. In our study, it is observed that the E_BD_ of the PMMA, PMP, and PMPMP is 535, 672, and 866 MV/m, respectively, as shown in Figure 4e.

Studies indicate that multilayered polymer films consistently exhibit higher electrical breakdown strength compared to their individual-film counterparts [42,43]. There are three important possible mechanisms working behind the ultra-high breakdown field of the PMPMP device: (a) the presence of multiple PMMA and 2D filler layers, (b) the addition of coverage of 2D Mica in a stratified geometry where two-dimensional surfaces of Mica are perpendicular to the applied electric field, and (c) the large surface area interaction between 2D fillers and polymers (interface polarization) between the 2D Mica in the nanocomposite.

During the electrical treeing process in a multilayer dielectric nanocomposite subjected to high electric fields, the propagation of electrical trees diverges at multiple interfaces along the in-plane direction [44,45]. Furthermore, the multilayer configuration restricts the free movement of carriers within the films, which in turn reduces the leakage current and enhances the breakdown strength [37,46,47,48,49]. The addition of second layers of Mica–PMMA (MP) onto the PMP device covers more space with 2D fillers by increasing the fillers on the surface while looking perpendicular to the film or along the direction of the applied electric field, resulting in enhanced breakdown strength of the PMPMP device. Therefore, the bilayer 2D Mica filler device (or PMPMP device) is resistant to high electric fields, exhibiting elevated E_BD_ and thereby enhancing U_d_. The inclusion of a second layer of 2D Mica also increases the interface between polymer and 2D Mica, leading to the enhancement of the film [10,26].

Our observations showed that 2D fillers oriented perpendicular to the direction of the electric field are more effective at preventing the expansion of electric breakdown paths through the nanocomposite, in contrast to randomly dispersed nanofillers [44,50]. The introduction of in-plane-oriented Mica nanofillers into the PMMA via mechanical exfoliation methods has contributed to improving the breakdown strength of the composite.

The calculated shape parameter (β) for the PMMA, PMP, and PMPMP is 7.2, 6.5, and 13.7, respectively, as shown in Figure 4e. The increase in energy density in multilayer polymer nanocomposites can be attributed to three major factors. (i) Increased dielectric constant: When these 2D nanofillers are integrated within the polymer layers, they can significantly increase the overall dielectric constant of the composite, as discussed earlier in the dielectric constant measurement. The maximum stored energy density (U) in a capacitor is proportional to the dielectric constant (ε) of the material. Therefore, a higher dielectric constant leads to a higher energy density at the same electric field strength. (ii) Improved polarization: The types of polarization that can occur in a polymer nanocomposite include electronic polarization, interfacial polarization, dipolar polarization, and ionic polarization [51]. Due to the substantial difference in dielectric constants between the PMMA and Mica, Maxwell–Wagner–Sillars interfacial polarization can develop at their interface. This phenomenon leads to an additional enhancement of interfacial polarization, and the overall polarization increases in the polymer nanocomposite, leading to an enhancement of discharged energy density. (iii) Improvement of breakdown strength: Multilayer polymer structures incorporated with insulating 2D filler render the electrical treeing process more effective and reduce the leakage current. This leads to an enhancement in breakdown strength and, hence, the energy density, of the nanocomposites.

## 3. Materials and Methods

In this study, 2D Mica flakes of varying thicknesses (single atomic layers to 30 nm thick) and sizes (1 m to 30 m) were exfoliated onto clean Si/SiO_2_. The thin layer of PMMA was spin coated on top of exfoliated Mica at 3000 rpm for 30 s followed by a short time annealing at 100 °C for 5 min. The PMMA-coated 2D Mica layer was etched out using 30 wt % KOH solutions and transferred onto a PMMA-coated ITO wafer to form a PMMA/2D Mica/PMMA (PMP) capacitor. We subsequently transferred a second 2D and PMMA layer using the same procedure onto the 1st PMMA/2D Mica/PMMA capacitor to form a PMMA/2D Mica/PMMA/2D Mica/PPMA (PMPMP) capacitor. Finally, the capacitor devices were annealed at 120 °C inside a vacuum chamber for 2 h. The distribution of the exfoliated 2D Mica flakes was visualized through an optical microscope. The thicknesses of the polymer and the 2D polymer nanocomposite were measured by a Veeco Dimension 3100 atomic force microscope. The temperature-dependent dielectric properties of 2D-nanocomposite films were measured by a HIOKI LCR tester (3522) at frequencies from 1 KHz to 7 MHz. The polarization and breakdown strength measurements were performed using a Radiant ferroelectric precession II instrument with a high-voltage power source (10 kV). Silver electrodes, with diameters ranging between 200 and 300 μm, were placed on top of the devices to serve as the top electrode (and capacitor area) in the experiments. We placed several devices throughout the film to discern the uniformity of the fillers’ film quality as well as their effects on dielectric and energy storage properties.

## 4. Conclusions

This study delved into the dielectric properties and electric polarization of PMMA/Mica nanocomposite fabricating multilayer capacitor devices. The incorporation of a 2D Mica nanofiller led to a remarkable increase in the dielectric constant, with improvements of approximately 15% and 54% observed for PMP and PMPMP structures, respectively, at room temperature. The pursuit of high discharged energy density and efficiency at a high operating electric field was witnessed in the multilayer nanocomposite PMP and PMPMP capacitor devices. Notably, the discharged energy density in the PMPMP reached approximately 38 J/cm^3^ at 825 MV/m, surpassing previously reported values for PMMA-based composite capacitors. The presence of interfacial polarization and insulating Mica filler within the polymer contributes to enhanced efficiency and increased breakdown strength. The enhancement of the dielectric constant with the dimensions of loss factor in the PMPMP device also justifies the high energy density and efficiency of the device. The exceptional capacitive and energy storage performance exhibited by these materials opens new avenues for advancing energy storage technologies, paving the way for more efficient and reliable energy storage solutions to meet the growing demands of modern society.

## Data Availability

The original contributions presented in this study are included in the article; further inquiries can be directed to the corresponding author.
